# Immune response profiling of HERV-W envelope proteins in multiple sclerosis: potential biomarkers for disease progression

**DOI:** 10.3389/fimmu.2024.1505239

**Published:** 2025-01-09

**Authors:** Stefano Ruberto, María I. Domınguez-Mozo, M. Angel Garcıa-Martınez, Davide Cossu, Leonardo A. Sechi, Roberto Alvarez-Lafuente

**Affiliations:** ^1^ Division of Microbiology and Virology, Department of Biomedical Sciences, University of Sassari, Sassari, Italy; ^2^ Environmental Factors in Degenerative Diseases Research Group. Instituto de Investigación Sanitaria del Hospital Clínico San Carlos (IdISSC), Madrid, Spain; ^3^ Department of Neurology, Juntendo University, Tokyo, Japan; ^4^ SC Microbiologia e Virologia, Azienda Ospedaliera Universitaria, Sassari, Italy

**Keywords:** multiple sclerosis, HERV-W, syncytin-1, biomarkers, immune response

## Abstract

**Introduction:**

The envelope proteins syncytin-1 and pHERV-W from the Human Endogenous Retroviral family ‘W’ (HERV-W) have been identified as potential risk factors in multiple sclerosis (MS). This study aims to evaluate both humoral and cell-mediated immune response to antigenic peptides derived from these proteins across different clinical forms and inflammatory phases of MS.

**Methods:**

Indirect enzyme-linked immunosorbent assay (ELISA) was employed to measure immunoglobulin G (IgG) responses to syncytin-1_env 486-500_ and pHERV-W_env 486-504_ peptides in MS patients. Discriminant analysis was used to assess whether clinical course prediction could be enhanced by integrating clinical variables with humoral response data against other MS-associated viral factors. Additionally, peripheral blood mononuclear cells from MS patients and healthy controls (HC) were analyzed for inflammatory responses following stimulation with these peptides.

**Results:**

MS patients exhibited significantly elevated antibody titers against -pHERV-W_env 486-504_ and syncytin-1_env 486-500_ compared to HCs, with the highest levels observed in progressive MS forms. Discriminant analysis accurately predicted the clinical course in 75.3% of the cases, with an 85% accuracy rate for progressive MS. *In vitro*, stimulation with pHERV-W_env 486-504_ led to a notable increase in pro-inflammatory cytokine production by CD4, CD8, and CD19 cells compared to syncytin-1_env 486-500_. _A_ strong correlation was found between pHERV- W_env 486-504_ induced cytokine production and EBV and CMV titers in MS patients.

**Discussion:**

These findings suggest that the pHERV-W envelope protein could be a valuable biomarker for monitoring peripheral inflammation in MS.

## Graphical Abstract

The pathogenic HERV-W envelope protein represents a potential biomarker for monitoring CNS inflammation in MS and may influence immune responses to other viruses, impacting inflammatory pathways and potentially altering disease progression and outcomes. (I) Retroviral transcripts from HERV-W family members, including Syncytin-1 and MS-associated retrovirus (pHERV-W), produce envelope proteins (env) that may contribute to MS pathology. (II) Anti- pHERV-Wenv IgG levels are significantly elevated in MS patients, correlating with disease progression. (III) When PBMCs are exposed to HERVW peptides, (IV) a notable increase in pro-inflammatory cytokine production by B and T-cells is observed, especially in comparison to syncytin-1. (V) Positive correlations are observed between proinflammatory cytokine production due to pHERV-Wenv exposure and anti-EBV antibody titters in patients with MS, suggesting a strong association between EBV and MS. (VI) This association could be mediated by EBV gp350 protein, which may activate neuropathogenic pHERV-W, leading to transcription of MSRV genes in PBMCs (8) and amplifying cytokines response. (VII) Conversely, a negative correlation is observed between proinflammatory cytokines produced by B and T cells upon pHERV-W exposure and anti-CMV IgG antibody levels, (VIII) suggesting that CMV seropositivity may modulate the inflammatory response by reducing cytokine production triggered by pHERV-W exposure. These findings indicate that pHERV-W plays a complex role in modulating immune responses in MS, with differential effects based on viral co-infections, such as EBV and CMV.

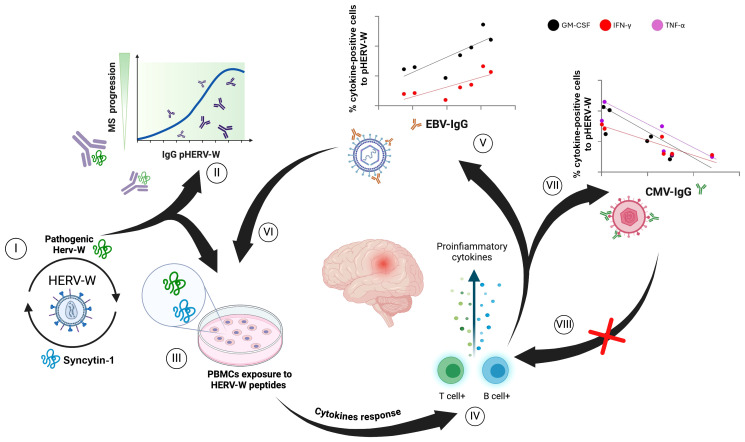

## 1 Introduction

Multiple sclerosis (MS) is a chronic inflammatory condition affecting the central nervous system (CNS), characterized by inflammation, demyelination, and neuronal damage (1). Despite being recognized for over a century, the precise etiology of MS remains unclear. Both genetic predisposition and several environmental factors are believed to play roles in the disease’s development (2). Among the viral agents potentially linked to MS onset and progression are *Epstein-Barr virus* (EBV), *Human herpesvirus-6A* (HHV-6A), and *Cytomegalovirus* (CMV) (3–5). Recent research suggests that human endogenous retroviruses of the W-family (HERV-W) may also be involved in MS progression (6).

About 8% of the human genome consists of endogenous retroviruses (HERVs), which are remnants of ancient viral infections (7). Among these, the W-family retroviruses, located on chromosome 7, produce a retroviral envelope (env) protein known as syncytin-1 encoded by endogenous retrovirus group W member 1 (ERVW-1), which is crucial for placental development (8). However, other genomic copies of W-family retroviruses have been linked to the pathogenesis of MS (9). Specifically, DNA sequences of MS-associated retroviruses (MSRVs) produce an envelope protein known as pHERV-W, which is implicated in MS pathology (10). At the protein level, a specific antibody targeting unique HERV-W proteins has not yet been developed (6). Furthermore, the origin of HERV-Wenv remains controversial. Unlike syncytin-1, MSRV has been hypothesized to represent an exogenous HERV-W, potentially a replication-competent but rare member, or an incompletely defective variant that occasionally recombines or is complemented within the HERV-W family (6, 8).

Research indicates that the env protein sequence of syncytin-1 and pHERV-W share 94% homology (11). Despite various single nucleotide polymorphisms throughout their sequences, the principal distinction is that MSRV contains an additional 12 nucleotides compared to ERVWE1, resulting in an extra four amino acids (His–Val–Leu–Gin). This high degree accounts for their similar properties, including their capacity to induce neurotoxicity and immune responses (6, 12). Extracellular sequences of the pHERV-W protein have been detected in the spinal fluid and blood of MS patients, suggesting a role in disease progression (6).

Elevated levels of pHERV-W have been observed in the brain and blood of MS patients (8), and this protein is considered potential markers for disease conversion and prognosis (13). Higher expression of these proteins in early disease stages of the disease may correlate with poorer outcomes, including increased disability, reduced treatment efficacy, and progression to more severe phases of MS (6, 8, 12, 14, 15). The pHERV-W can activate the body’s innate immune system by interacting with toll-like receptor (TLR) 4 and CD14 coreceptors, which are implicated in the pathogenesis of MS (6, 12, 16, 17). This activation stimulates the production and release of proinflammatory cytokines, including TNF-α, IL-12 and IFN-γ, which contribute to an exacerbated immune response (18). Consequently, the pHERV-W antigen may elicit a more intense immunological response than syncytin-1, potentially worsening the inflammatory response in MS patients (11).

Research has demonstrated variations in immune responses to HERVs among individuals with relapsing-remitting MS, depending on whether they are in acute disease phases or stable phases (19, 20).

This study aims to investigate the humoral immune response to specific antigenic peptides derived from syncytin-1 and pHERV-W, as well as and other MS-related viral antigens, such as EBV, HHV-6A/B and CMV. This study will compare these responses in healthy controls (HCs) and MS patients across different disease phases, including remission, both stable and acute, and progressive phases. Additionally, the cell-mediated inflammatory response, focusing on major proinflammatory cytokines, will be evaluated in MS cells exposed to pHERV-W_env 486-504_ and syncytin-1_env 486-500_ epitopes. These regions were selected based on analysis using the Immuno Epitope Database and Analysis Resource (IEBD), specifically targeting the extra four amino acids present in the envelope protein of pHERV-W. The IEDB software predicts which protein regions likely to be recognized as epitopes in the context of both T and B-cell responses.

The investigation will also evaluate T and B-cell-mediated responses to these epitopes to assess if similar T and B-cell clones can recognize the studied peptides.

## 2 Materials and methods

### 2.1 Population study

A total of 324 MS patients and 175 age/sex-matched HCs were recruited from the Hospital Clínico San Carlos in Madrid, Spain. Recruitment took place from April 2010 to May 2020, with all participants providing informed consent via forms approved by the Clinical Research Ethics Committee. The MS cohort included patients with relapsing-remitting MS (RR-MS) either active (RR-AMS, with relapses and/or evidence of MRI activity within two weeks of symptoms onset) or stable MS (RR-SMS, defined by the absence of disease activity in the 3 months prior to sample collection) primary progressive MS (PP-MS), and secondary progressive MS (SP-MS) (McDonald criteria) (21). Demographic, clinical, and radiological data were collected from medical records or during study inclusion, as summarized in **Table 1**. Participants had not received disease-modifying therapy for at least one month before the blood collection. Specifically, RR-AMS samples were collected prior to corticosteroid administration, and some untreated patients were sampled after switching treatments and completing to another and a washout period.

**Table 1:** Demographic and clinical data of MS patients.

Parameter	Values				
	HC	RR-SMS	RR-AMS	PP-MS	SP-MS
**No.**	175	149	62	61	52
**Sex † (no. of men/women)**	44/131	43/106	15/47	31/30	26/26
**Age (yr) † † [median ± SD]**	48 ± 6	47 ± 9	50 ± 8	57 ± 10	59 ± 8
**EDSS ††† [median (range)]**	–	2.1 (0-2)	2.5 (0-5.5)	5 (0 ± 8.5)	5.3 (2.5-8.5)
**Age (yr) at MS onset [median ± SD]**	–	30 ± 9	28 ± 8	39 ± 11	33 ± 10

†According to gender distribution, the following statistically significant differences were found between HC *vs* RR-SMS (*p* = 0.45); HC *vs* RR-AMS (*p* = 0.88); HC *vs* PP-MS (*p* = 0.0002); HC *vs* SP-MS (*p* = 0.0007). Chi-square test. *p* ≤ 0.05 was considered statistically significant.

††Based on the age distribution, the following statistically significant differences were found between the two groups. HC *vs* RR-SMS (*p* = 0.0003); HC *vs* RR-AMS (*p* = <0.0001); HC *vs* PP-MS (*p* = <0.0001); HC *vs* SP-MS (*p* = <0.0001). Mann Whitney test. *p* ≤ 0.05 was considered statistically significant.

†††Expanded Disability Status Scale

### 2.2 Blood processing

Peripheral blood (20 mL) was collected from each participant: 10 ml into dry tubes for serum collection and 8 ml into CPT™ tubes (Cell Preparation Tube; Becton Dickinson, Franklin Lakes, NJ, USA) for PBMC isolation. Serum was obtained by centrifugation at 900 x g for 15 min at room temperature (RT) and stored at -80°C until analysis. PBMCs were isolated using Ficoll-Paque™ gradient centrifugation at 900 x g for 30 min at RT. After centrifugation, the cells were washed with saline buffer and centrifugated again at 400 x g for 10 min at RT, discarding the supernatant. The cell pellet was then resuspended in a mixture of 10% dimethyl sulfoxide (DMSO) and 1 ml of fetal bovine serum and stored temporarily at -80° in a MrFrostie container before being transferred to liquid nitrogen (-196°C).

### 2.3 Peptides

Synthetic peptides with a purity > 95% were sourced from LifeTein (South Plainfield, NJ, USA) and SynPeptide Co Ltd (USA). These peptides were dissolved in DMSO at a concentration of 10 mM and stored at -80°C. The peptides included syncytin-1_env 486–500_ (UniProt accession no. Q9UQF0) with the sequence QMEPKMQSKTKIYRR, and pHERV-W_env 486–504_ (UniProt accession no. Q991W9) with the sequence QIVLQMEPQMQSMTKIYRG.

### 2.4 Indirect Enzyme-linked immunosorbent assay

Serum antibodies against retroviral peptides were detected using an indirect ELISA to measure immunoglobulin G (IgG). To perform the peptide-based indirect ELISA, ninety-six-well immune-plates were coated with 50 µL per well of either the syncytin-1_env 486-500_ or pHERV-W_env 486–504_ peptides, both at equimolar concentrations of 10 μM, diluted in ELISA coating buffer at 0.05 M of carbonate–bicarbonate (pH 9.5, Sigma-Aldrich, St. Louis). The plates were incubated overnight at 4°C. The following day, the microplates were incubated for 1 hour at RT (25°C) using a blocking solution of 1% non-fat dried milk (Sigma-Aldrich, St. Louis, MO, USA) in tris-buffered saline (TBS). After blocking, the plates were washed twice with TBS containing 0.05% Tween-20 (TBS-T). Sera samples, diluted 1:10 dilution in the blocking solution, were added in duplicate wells and incubated for 2 hours at RT. The plates were then washed with TBS-T. Subsequently, the plates were incubated with 100 µL per well of alkaline phosphatase-labeled, Fc-specific, anti-human IgG polyclonal antibodies (1:5000, Sigma-Aldrich, St. Louis, MO, USA), for 1 hour at RT. After another wash, the wells were incubated with 200 µL per well of milli-Q water containing p-nitrophenyl phosphate (Sigma-Aldrich, St. Louis, MO, USA) for 30 minutes at RT in the dark. Optical density was measured at 405 nm using a SpectraMax Plus 384 microplate reader (Molecular Devices, Sunnyvale, CA, USA). Negative control wells were included on each plate, and the mean optical density value from these wells was subtracted from all other data points. Results were normalized against a positive control serum included in all experiments.

### 2.5 Herpesvirus 6A/B, Epstein-Barr virus and Cytomegalovirus by automated ELISA

Serum samples were assessed using commercial tests: anti-EBNA-1 and anti-VCA IgG (Trinity Biotech, USA), anti-CMV IgG (Vircell, USA), and anti-HHV-6A/B IgG and IgM (Vidia, Ltd., Czech Republic). The analysis was performed using an automated ELISA processing system (DS2, Dynex Technologies, USA). Results were reported in artificial units (AU), calculated by multiplying the index value by 10 (where the index value is the sample absorbance divided by the cut-off value) (22, 23). Each sample was tested in duplicate. Values below 11 AU were considered negative, while samples with values between 9 and 11 AU were reanalyzed.

### 2.6 Cell culture

PBMCs were thawed from liquid nitrogen and washed three times (400 x g, 10 min, RT) in complete medium consisting of RPMI-1640 (Roswell Park Memorial Institute medium; Merck, Darmstadt, Germany), 10% fetal bovine serum, 0.5% streptomycin-penicillin, and 1% L-glutamine. Cell viability was assessed using trypan blue exclusion staining, and cell counting was performed with a Neubauer chamber. The cells were then seeded into a 12-well cell culture plate at a density of 2 x 10^6^ PBMCs per ml and cultured overnight in an incubator at 5% CO_2_ and 37°C. The following day, PBMC cultures were washed in complete medium (400 x g, 10 min, RT), counted to assess viability and recovery, and resuspended at a density of 2 x 10^6^ per ml.

### 2.7 Antigen stimulation

Cells from 14 MS patients (7 RR-SMS and 7 PP-MS) and 9 HCs were distributed into 5 tubes, each containing 5 x 10^5^ cells. The experimental conditions included negative control (RPMI to 10% FBS), positive controls (phorbol 12-myristate 13-acetate at 50ng/ml and calcium ionophore at 0.75µg/ml (Sigma Aldrich), vehicle control (DMSO at 0.75%), and two antigen stimulations with syncytin-1_env 486–500_ and pHERV-W_env 486–504_ peptides, both at 75µM, with costimulatory antibodies anti-CD28/CD49d (5µg/ml) (BD Biosciences, San Diego, CA, USA). The cells were incubated at 5% CO_2_ and 37°C for 2 h. During the last 6 hours of culture, brefeldin A (BFA) (10 µg/ml) and monensin (0.7 µg/ml) (BD Biosciences, San Diego, CA, USA) were added. After incubation, cell cultures were washed with saline buffer (PBS) and centrifugated at 300 x g for 5 min at RT, discarding the supernatant.

### 2.8 Cell surface and intracellular cytokine staining

Cell pellets were resuspended in 1 mL of culture medium and incubated with a mix of fluorochrome-conjugated antibodies specific for cell surface markers. The staining was performed for 20 min at 4°C in the dark. The antibodies used included: CD45-V500, CD3-BV421, CD8-APC-H7 and CD19-PE-Cy7 (BD) were added. Following incubation, the cells were washed with PBS and centrifugated at 500 g for 5 min at RT. The supernatant was discarded.

Cells were fixed and permeabilized using Cytofix/Cytoperm (BD Biosciences, San Diego, CA, USA) following the manufacturer’s instructions. After fixation, cells were washed with PERM-WASH buffer and centrifugated at 500 x g for 5 min at RT, discarding the supernatant. Intracellular staining was performed with fluorescein isothiocyanate (FITC) anti-IFN-γ, allophycocyanin (APC) anti-IL-17, Peridinin chlorophyll (PercP-Cy5.5) anti-TNF-α, and Phycoerythrin (PE) anti-GM-CSF. Staining was carried out at 4°C for 20 minutes in the dark. After extensive washing with PERM-WASH buffer, cells were resuspended in 300 μL of 1% paraformaldehyde in PBS. Samples were analyzed using FACS-Canto II 8-color flow cytometer (BD) and data were processed with CytExpert software (Beckman Coulter, USA), acquiring 150.000 events for sample. Analysis strategies for lymphocyte subpopulations are detailed in **Supplementary Figure 1**.

### 2.9 Statistical analysis

Categorical variables were expressed as percentages, while normally distributed numerical variables were reported as mean ± standard deviation, and non-normally distributed variables as median (25th-75th percentile). ELISA results between patients and HCs were compared using the non-parametric Mann–Whitney’s U-test. The accuracy of the ELISA assay was assessed using receiver operator characteristic (ROC) curves. The coefficient of variation (CV) for intra-assay was capped at 10%. Outliers were identified by the Interquartile Range (IQR) method, with a no-negative constant of three. Cut-off values for positivity in each test were set at 95% specificity, and sensitivity was calculated accordingly. The Shapiro-Wilk test was performed to evaluate the normality of data distribution. Non-parametric tests, including Mann-Whitney, Kruskal-Wallis and Spearman correlation, were employed for multiple comparisons and correlation analyses. Discriminant analysis was used to study population distribution, followed by multivariate Hotelling T^2^ analysis. This statistical method identifies which variable discriminates between groups based on quantitative and qualitative measures. The method extrapolates n-1 discriminant functions (DF), where n is the number of groups to be discriminated, which are linear combinations of the selected original quantitative variables. These derived functions can be used to calculate a set of discriminant scores that are used to predict the status of a new observation. Therefore, the first discriminator function, known as DF1, maximizes the variance between the variable’s values. On the other hand, the second discriminator function, known as DF2 which is orthogonal to DF1, maximizes the residual differences between values of these variables. The model parameters are the *eigenvalues*, a measure of the variance in the variable for each function; the *Wilks’ lambda*, an index of discriminating power within the range of 0 to 1 (the lower the value, the higher the discriminating power) and the canonical correlation a measure of the associations between the groups formed by the sets of variables and the DF, where the higher this value, the stronger the correlation between the groups and the DFs.

Statistical significance set at *p*-value of 0.05. Data analysis was performed using GraphPad Prism software (versions 8.0/9.0, San Diego, CA, USA) and SPSS software package (version 28.0, SPSS, Inc).

## 3 Results

### 3.1 Elevated anti-pHERV-W_env 486-504_/syncytin-1_env486-500_ IgG response in MS patients: correlation with disease progression and disability status

The peptide ratio was calculated to investigate the potential imbalance between the physiological syncytin-1 and the pathological pHERV-W. Antibody production against the same epitope varies among individuals due to factors like genetics. Analyzing the ratio of antibodies against pHERV-W and syncytin-1 two proteins with different roles, one pathogenic and the other physiological, allows us to account for this variability and to correct the individual heterogeneity in antibody production. Therefore, this ratio reflects the immune system’s response to two distinct antigens, and any imbalance between these antibodies could indicate that one antigen is driving a stronger immune reaction. Such shifts in immune response may provide understanding of the mechanisms and their potential role in disease pathogenesis.

**Figure 1:**
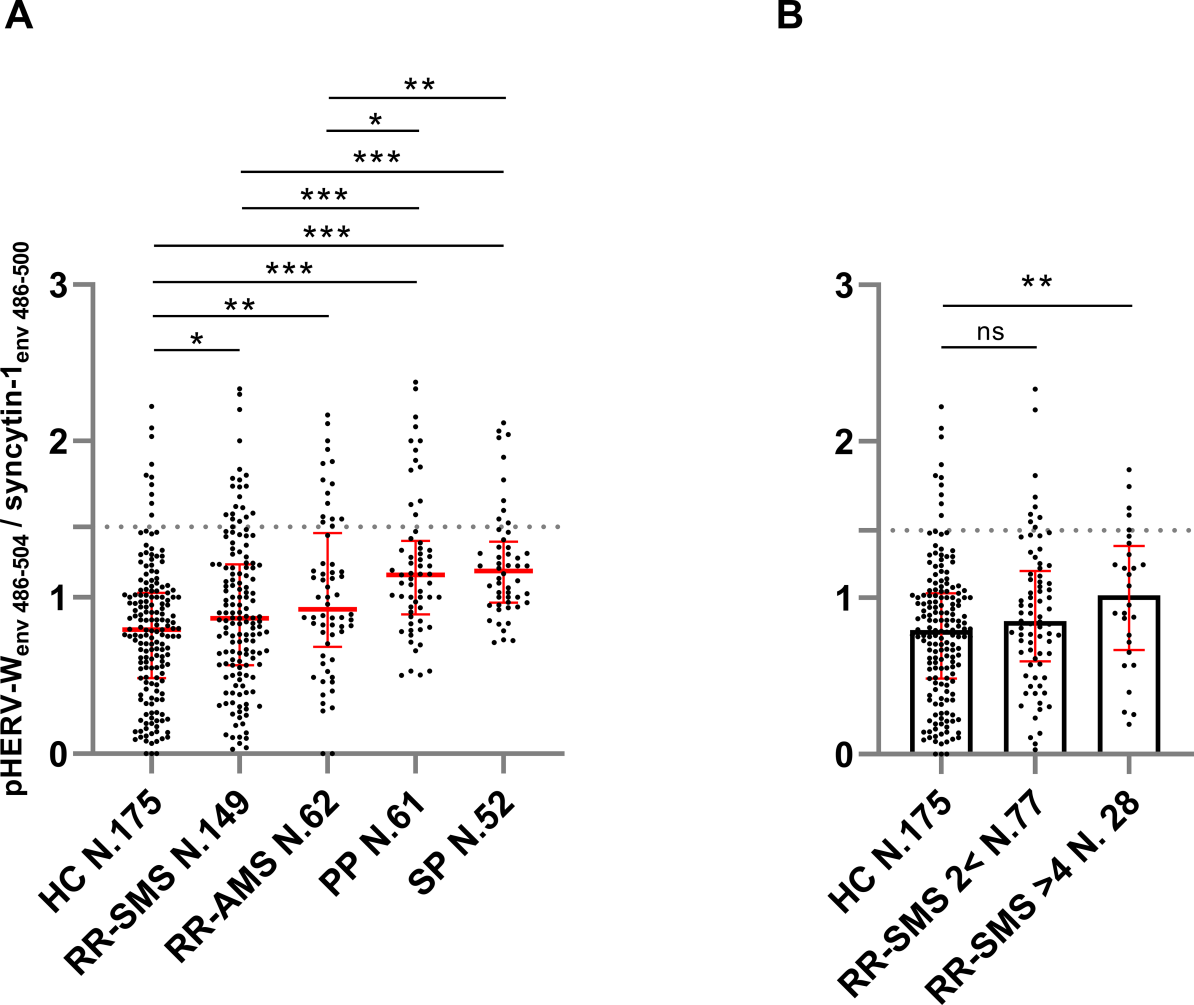
Ratio of the antibody titers against pHERV-W_env 486–504_ *vs* syncytin-1_env 486–500_, detected by ELISA in sera of MS patients and healthy controls (HCs). **(A)** Antibody ratio titers in patients with stable RR-MS (SMS), acute RR-MS (AMS), PP-MS, SP-MS and HCs; the median with interquartile range in red lines. **(B)** Antibody ratio titers in sera of patients with stable RR-MS (SMS) with EDSS score < 2 and > 4, and healthy controls (HCs); the median (column height) with interquartile range in red line. Graphs displaying the cutoff values (dashed lines) for the positivity applied. Each dot represents the titer of a patient. Shapiro–Wilk, Mann–Whitney test and Kruskal–Wallis with Dunn’s test. *p* ≤ 0.05 was considered statistically significant. *p ≤ 0.05; **p ≤ 0.01; ***p ≤ 0.001, ns not significant.

The ratio of anti-pHERV-W_env 486-504_/anti-syncytin-1_env486-500_ IgG response in serum was higher across all categories of MS patients compared to HCs. Specifically, the ratio levels were as follows: RR-SMS 0.87, (0.57-1.21); RR-AMS 0.92 (0.72-1.35); PP-MS 1.14 (0.91-1.35); SP-MS 1.17 (0.973-1.33); HCs 0.79 (0.48–1.02) (**Figure 1**).

In progressive forms of MS (PP-MS, SP-MS), there was a notably higher pHERV-W_env 486-504_/syncytin-1_env486-500_ IgG response compared to RR-MS, including both RR-SMS and RR-AMS phases (**Figure 1A**). Furthermore, RR-SMS patients with an EDSS > 4 showed higher pHERV-W_env 486-504_/syncytin-1_env486-500_ IgG-antibody titers (1.02, 0.70-1.29) than HCs. However, no significant difference was observed in the humoral response between RR-SMS patients with an EDSS < 2 (0.85, 0.61-1.15) and HCs (**Figure 1B**). Although it exists an imbalance with regard to demographic variables (**Table 1**), no significant correlation was observed between the antibody response to both epitopes and variables such as age or gender.

### 3.2 Epstein-Barr virus and Cytomegalovirus IgG and Herpesvirus *6A/B* IgM/G ELISA in MS and HCs

Qualitative titer-based ELISA showed a higher prevalence of anti-EBV/EBNA-1 IgG in RR-SMS (95%), RR-AMS (100%), and PP-MS (97%) compared to SP-MS (92%) and HCs (86%). There was no difference in the prevalence of anti EBV/VCA IgG among all groups. Conversely, a lower prevalence of anti-CMV IgG was detected in RR-SMS (59%) compared to RR-AMS (69%), PP-MS (69%), SP-MS (68%), and HCs (74%).

**Table 2** presents the quantitative analysis of HHV-6A/B IgM and IgG, along with EBV/EBNA-1, EBV/VCA, and CMV IgG antibody titers, highlighting significant differences between MS patients and HCs. RR-MS patients exhibited higher EBNA-1 and HHV6A/B IgG-IgM titers than HCs, while EBV/VCA titers were significantly elevated in RR-AMS, PP, and SP patients. Notably, lower CMV-IgG levels were observed in RR-SMS patients compared with HCs. Furthermore, graphical representation of this distribution is illustrated in **Supplementary Figure 2**. Additionally, moderate positive correlations were detected between pHERV-W_env 486-504/_syncytin-1_env486-500_ and HHV-6A/B IgM^+^ (N =18, *r* = 0.6, *p* = 0.009) and IgG^+^ (N = 44, *r* = 0.3, *p* = 0.05) levels in PP-MS patients (data not shown).

**Table 2:** Quantification of antibody titters for HHV-6A/B IgM and IgG, EBV/EBNA-1, EBV/VCA, and CMV IgG in patients and controls.

Mean ± SD Median (IQR)		HC	RR-SMS	RR-AMS	PP-MS	SP-MS
**HHV-6A/B**	IgM	7.9 ± 6.2 6.1 (3.7-10.3)	7.2 ± 7.8 4.9 (3.5-7.8)	9.7 ± 12.4 5.5 (2.6-10.9)	7.3 ± 6.6 4.5 (1.9-11.3)	9.3 ± 10.1 6.1 (2.7-12.0)
	IgM+	16.5 ± 6.6 13.8 (12.2-17.6)	27.1 ± 15.9* 21.1 (16.4-37)	23 ± 14.9 19.4(17.1-25.5)	15.2 ± 4.4 14.7 (10.8-18.3)	19.1 ± 10.1 16.4 (12.0-23.1)
**HHV-6A/B**	IgG	23.2 ± 12.7 20 (13.4-33.5)	26.0 ± 16.1 24 (15-36.5)	23.6 ± 10.8 21.4 (17.4-31.7)	21.3 ± 15.6 16.9 (9.3-29.6)	16.6 ± 7.7* 15.8 (11.6-19.6)
	IgG+	26.2 ± 11.6 23.9 (15.7-35.3)	30.4 ± 14* 28.6 (18.6-38)	24.2 ± 10.1 21.7 (17.9-32.4)	26.5 ± 14.1 20 (16.2-38.7)	18 ± 7.1* 17.2 (14.5-20.8)
**EBV/EBNA-1**	IgG	18.5 ± 6.9 19.7 (15-23.2)	22.4 ± 6.2* 22.9 (20-25.8)	22.2 ± 5.1* 21.8 (18.2-25.9)	19.6 ± 6.6 19 (15.9-22.9)	20.7 ± 7.17 20.4(18.0-24.9)
	IgG+	20.6 ± 4.9 20.2 (17.1-23.6)	23.3 ± 4.5* 23 (20.7-25.9)		20.1 ± 6.1 19.2 (16.4-23.4)	22.1 ± 5.4 21 (18.3-25.2)
**EBV/VCA**	IgG	43.3 ± 14.8 45.2 (35.5-53.1)	46.7 ± 12.8 47.6 (40.1-54.1)	52.9 ± 12.2* 55.5 (45.5-61.5)	55.1 ± 12.2* 56.2 (47-63.3)	49.3 ± 11.7* 49.8 (44.4-58.5)
	IgG+	44.8 ± 12.9 45.7 (37.8-53.4)	47.3 ± 11.8 47.7 (40.5-54.3)			
**CMV**	IgG	21.4 ± 12.7 24.4 (10.4-29.1)	17.9 ± 15.9* 18.6 (1.5-29.4)	18.2 ± 13.1 20.3 (2.3-28.5)	22.1 ± 15 26.5(4.6-32.1)	19.6 ± 14.6 25.3(2.3-30.9)
	IgG+	27.8 ± 7.2 27.1 (23-30.9)	28.8 ± 11.12 26.6 (21.2-33.7)	25.9 ± 8.1 26.3 (19.6-31.3)	30.9 ± 8.5 30.2 (25.9-36.6)	28.5 ± 7.7 29.2 (25.4-31.8)

Data are presented as mean +/- SD and median (IQR). The tables include the level of total IgG (or IgM) antibodies in patients and controls, as well as the proportion of IgG (or IgM) level above the established cut-off (IgG+/IgM+). Statistical analysis was performed using the Kruskal–Wallis and Mann–Whitney test. *Indicate significant differences compared to HCs.

### 3.3 Multivariate discriminant analysis of clinical and immunological variables differentiating RR-MS, SP-MS, and PP-MS

A Multivariate Discriminant Analysis (MDA) was conducted to determine the factors that most effectively distinguish between the RR-SMS, SP-MS, and PP-MS groups. The analysis considered ten categorical and independent variables, encompassing both clinical and immunological factors. **Table 3** presents the model’s parameters, including the *eigenvalues*, *variance*, *canonical correlation*, and *Wilks’ Lambda* value, along with the matrix structure coefficients that indicate the correlations between each categorical variable and the discriminant functions (DFs). The DF1 accounted for 87.3% of the total variance, demonstrating a strong canonical correlation value of 0.737 and a *Wilks’ Lambda* value of 0.389, while the DF2 accounted for the remaining 12.7% of the variance.

**Table 3:** Matrix structure coefficients, percentage of variance, Eigenvalues, canonical correlations, and Wilks’ Lambda of the classification model of MS patients.

	Function	
	1	2
***Variables***	–	–
**EDSS**	0.785*	-0.070
**Age**	0.551*	-0.159
**pHERV-W/syncytin-1**	0.314*	0.044
**HHV6A/B IgG**	-0.245*	0.234
**EBV/EBNA-1 IgG**	-0.174*	-0.094
**Disease Duration (month)**	0.302	-0.726*
**Age Disease (Onset)**	0.333	0.537*
**EBV/VCA IgG**	0.229	0.297*
**CMV IgG**	0.067	0.168*
**HHV6A/B IgM**	0.087	-0.118*
***Parameters of the Model***	–	–
**% of variance**	87.3	12.7
***Eigenvalues***	1.19	0.17
**Canonical correlation**	0.737	0.385
**Wilks’ Lambda**	0.389	0.852

The discriminant power of each categorical variable in the discriminant functions is shown.

*Largest absolute correlation between each variable and any discriminant function

According to MDA, **Supplementary Table 1** (Supplementary Data) summarizes discriminant coefficients used to assess the relative importance of the dependent variables. Standardized discriminant coefficients are provided to compare the relative significance of the clinical and immunological variables in predicting the dependent outcomes. Thus, variables with higher absolute values indicate a greater contribution to the discriminating power of the model.

**Figure 2:**
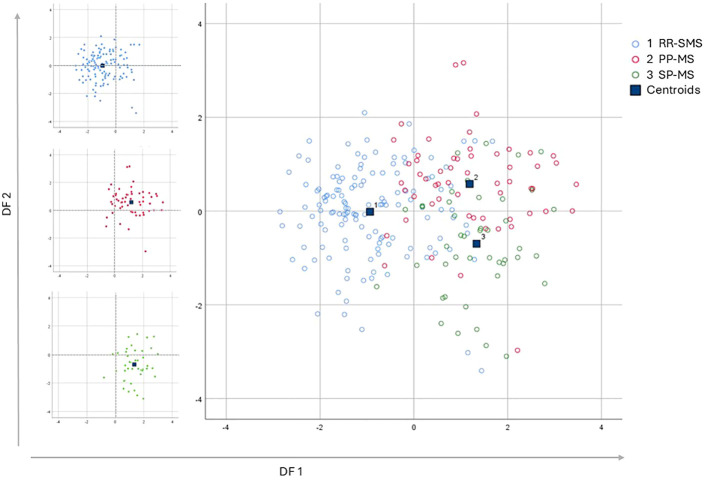
Multi discriminant analysis scatter plot of discriminant function 1 *vs* discriminant function 2, which explain the 87.3% and the 12.7% respectively of the total variance. The graphs display the distribution of the three populations, represented by the variance of the discriminant functions derived from the investigation of the independent variables. On the Left side the three scatter plot of the populations graphed separately; the RR-SMS (blue; n=135), PP-MS (red; n=57) and SP-MS (green; n=44). On the right side the three populations combined. RR-SMS: relapsing-remitting stable phase (1), PP-MS: primary progressive (2) and SP-MS: secondary progressive (3). Centroid: represented by blue square, highlight the center of mass of each population density, DF: discriminant function.

The application of DF1 and DF2 achieved a correct classification rate of 74.2% for the original cases. Cross-validation of these functions resulted in a classification rate of 71.2%. **Table 4** shows the classification matrix, illustrating how accurately the model categorized the participants groups in the study. The highest classification accuracy was achieved for RR-SMS patients at 81.5%, while the lowest was for the PP-MS patients at 63.2%. The greatest challenge in classification was observed between the primary and secondary progressive forms. The discriminative power of the two functions is illustrated in the scatter plot of **Figure 2** for DF1/2. DF1 effectively distinguished between progressive forms (PP-MS and SP-MS) to RR-SMS, while DF2, with lower discriminative power, differentiate between primary and secondary progressive forms. Multivariate Hotelling’s T2 analysis, which employs the two discriminant functions, assessed the statistical similarity among patient groups. The Hotelling’s T2 test revealed significant differences between RR-SMS (blue) and PP-MS patients (red) (*p* < 0.001, centroids distance 2.2) as well as between RR-MS and SP-MS (green) (*p* < 0.001, centroids distance 2.3). Additionally, PP-MS and SP-MS patients exhibited significant differences (*p* < 0.001, centroids distance 1.3).

**Table 4:** Classification matrix in MS patients.

Type of classification	Group	Predicted group membership			Total
		RR-SMS	PP-MS	SP-MS	
**Original**	RR-SMS	110 (81.5%)	14 (10.4%)	11 (8.1%)	135 (100%)
	PP-MS	8 (14%)	36 (63.2%)	13 (22.8%)	57 (100%)
	SP-MS	2 (4.5%)	13 (29.5%)	29 (65.9%)	44 (100%)
**Cross-validated**	RR-SMS	108 (80%)	16 (11.9%)	11 (8.1%)	135 (100%)
	PP-MS	9 (15.8%)	34 (59.6%)	14 (24.6%)	57 (100%)
	SP-MS	5 (11.4%)	13 (29.5%)	26 (59.1%)	44 (100%)

### 3.4 Differential cytokine responses in T and B cells to pHERV-W and syncytin-1 peptides in MS

**Figure 3:**
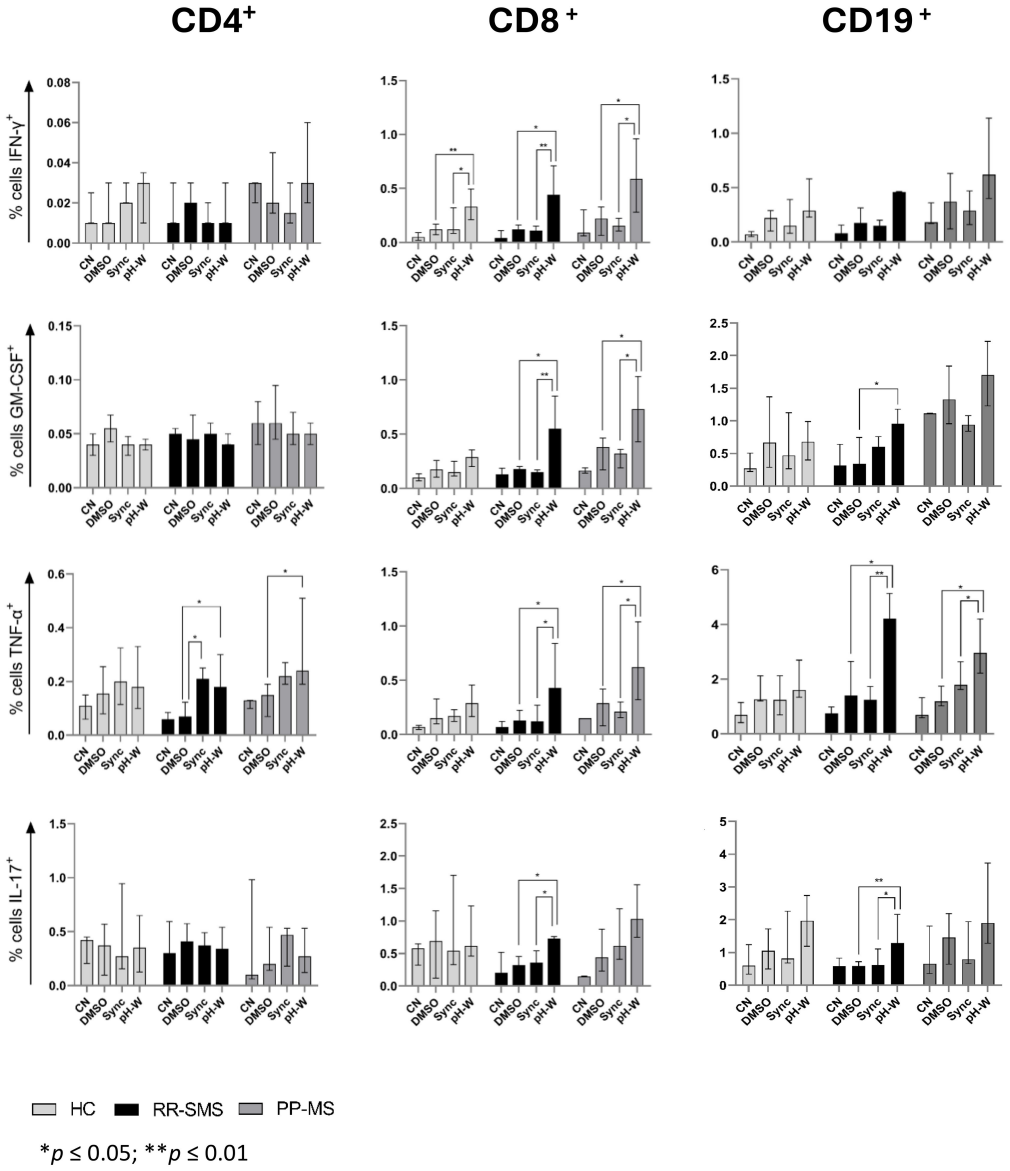
Detection of CN, DMSO (vehicle-control), Sync (syncytin-1_env 486-500_), and pH-W (pHERV-W_env 486-504_)-specific cytokine-positive T/B-cells by intracytoplasmic cytokine expression assay. Percentage of cytokine-positive CD4 (left), CD8 (center) and CD19 (right) in HC (n= 9), RR-SMS (n=7) and PP-MS (n=7). The figures display the median with interquartile range. Mann–Whitney test and Kruskal–Wallis with Dunn’s test. *p* ≤ 0.05 was considered statistically significant. CN, negative control; DMSO, Dimethyl sulfoxide; Sync, syncytin-1_env 486-500_; pH-W, pHERV-W_env 486-504_; IFN-γ, interferon-gamma; GM-CSF, Granulocyte-macrophage colony-stimulating factor; TNF, tumor necrosis factor-alpha; IL, interleukin; HC, healthy control; RR-SMS, relapsing-remitting stable phase; PP-MS, primary progressive.

**Figure 4:**
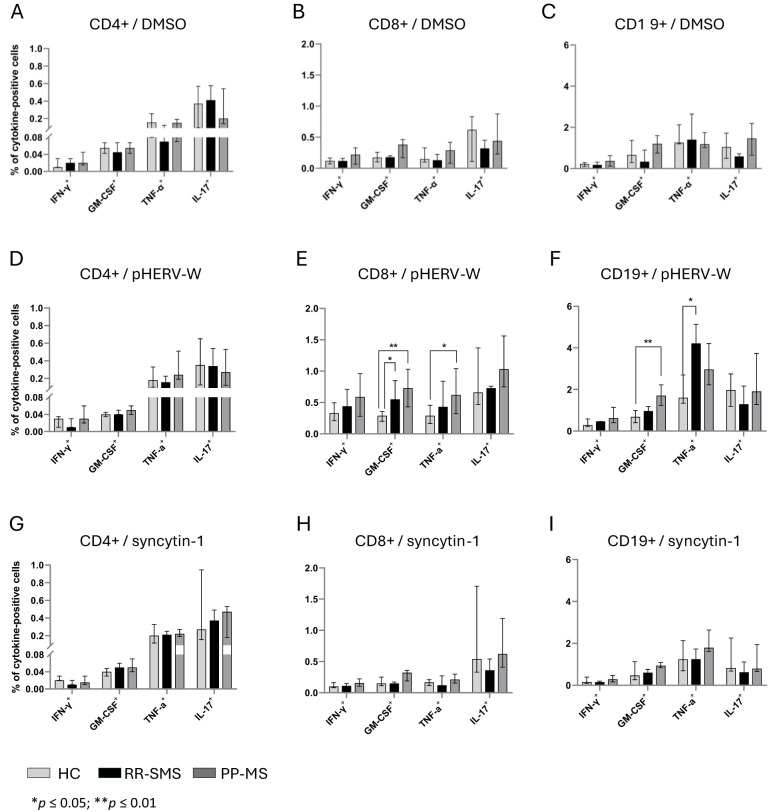
Cytokine-positive CD4, CD8 and CD19 detection in PBMCs of HC, RR-SMS and PP-MS. Percentage of CD4^+^-CD8^+^/T and CD19^+^/B cells secreting IFN-γ, GM-CSF, TNF-α and IL-17 after 8 h incubation with DMSO (vehicle control) **(A–C)**, pHERV-W_env 486-504_ **(D–F)** or syncytin-1_env 486-500_ **(G–I)**. Mann–Whitney test and Kruskal–Wallis with Dunn’s test. *p* ≤ 0.05 was considered statistically significant. IFN-γ, interferon-gamma; GM-CSF, Granulocyte-macrophage colony-stimulating factor; TNF, tumor necrosis factor-alpha; IL, interleukin; HC, healthy control (n=9); RR-SMS, relapsing-remitting stable phase (n=7); PP-MS, primary progressive (n=7).

The percentage of CD4+ cells secreting IFN-γ, GM-CSF, TNF-α, and IL-17 was measured in MS patients and HCs (**Figure 3**). In RR-SMS patients, a significant increase in TNF-α expression was observed in CD4+ cells exposed to syncytin-1_env 486-500_ and pHERV-W_env 486-504_ compared to DMSO control. In PP-MS patients, a significant increase in TNF-α expression was observed in CD4+ cells exposed to pHERV-W_env 486-504_ compared to DMSO. In contrast, none of the tested conditions induced cytokines production in CD4+ cells from HCs (**Figure 3**). No significant difference in cytokines production by CD4+ cells exposed to DMSO (vehicle control), pHERV-W_env 486-504_ or syncytin-1_env 486-500_ were found between HCs, RR-SMS and PP-MS groups (**Figures 4A, D, G**).

The same experimental conditions applied to CD4 T-cells were assessed for CD8+ T cells. PBMCs from MS patients stimulated to pHERV-W _env 486-504_ showed that CD8+ T–cells expressed cytokines at frequencies greater than 0.5% and 0.4% compared to DMSO and syncytin-1 _env 486-500_, respectively (**Figure 3**). In RR-SMS patients, exposure to pHERV-W_env 486-504_ resulted in a significant increase in the expression of all studied cytokines in CD8+ cells compared to DMSO and syncytin-1_env 486-500_. Similarly, PP-MS patients exhibited significantly higher levels of IFN-γ, GTM-CSF and TNF-α cytokines in CD8+ cells exposed to pHERV-W_env 486-504_ compared to DMSO and syncytin-1_env 486-500_ (**Figure 3**). In HCs, significant differences were observed only in IFN-γ production by CD8+ cells exposed to pHERV-W_env 486-504_ compared to DMSO and syncytin-1_env 486-500_. Furthermore, cytokines levels produced by CD8+ cells exposed to pHERV-W_env 486-504_ were significantly different between HCs and both RR-SMS and PP-MS patients (**Figure 4E**). Notably, CD8+ T-cells from PP-MS patients exhibited a substantial increase in GM-CSF (+0.42%; *p* < 0.01) and TNF-α (+0.34%; *p* = 0.03) levels compared to HCs, while RR-SMS patients had a significant increase in GM-CSF levels (+0.30%; *p* = 0.02) compared to HCs. No significant difference in cytokines production by CD8+ cells exposed to DMSO (**Figure 4B**) or syncytin-1_env 486-500_ (**Figure 4H**) were found between HCs, RR-SMS and PP-MS groups.

Intracellular cytokines levels in CD19 B-cells exposed to syncytin-1_env 486-500_ and pHERV-W_env 486-504_ were analyzed (**Figure 3**). In RR-SMS patients, a significant increase in TNF-α and IL-17 expression were observed with pHERV-W_env 486-504_ compared to DMSO and syncytin-1_env 486-500_, along with an increase in GM-CSF compared to DMSO (**Figure 3**). PP-MS patients also showed significant increases in TNF-α expression with pHERV-W_env 486-504_ compared to both DMSO and syncytin-1_env 486-500_. No significant cytokine increases were observed in HCs. Differences in cytokine levels between HCs, RR-SMS and PP-MS were significant with pHERV-W_env 486-504_ (**Figure 4F**). PP-MS patients had notably higher GM-CSF (+1.03%; *p* < 0.01) and RR-SMS patients had a higher TNF-α (+1.87%; *p* = 0.05) compared to HCs. No significant difference in cytokines production by CD19+ cells exposed to DMSO (**Figure 4C**) or syncytin-1_env 486-500_ (**Figure 4I**) were found between HCs, RR-SMS and PP-MS groups.

**Figure 5:**
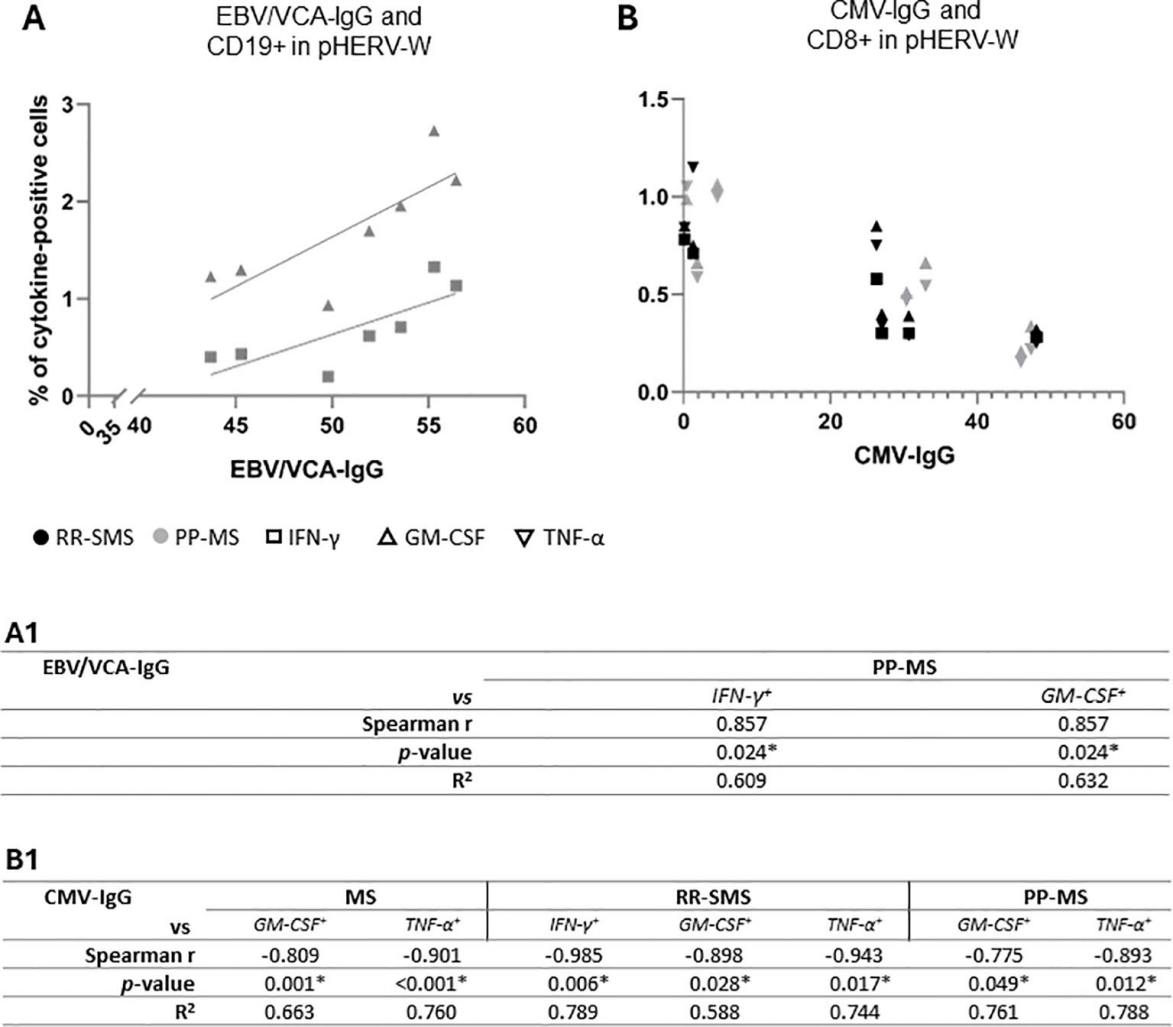
Scatter plot showing the correlations between the EBV/VCA-IgG **(A)** and CMV-IgG **(B)** antibody titers and the pHERV-W cytokine-positive CD-19 and CD-8, respectively, in RR-SMS (n=7) and PP-MS (n=7) patients. Below, non-parametric Spearmen correlation coefficient (*r*) and R2 from simple linear regression analysis, both for the entire population (MS) and the two MS clinical forms (RR-SMS and PP-MS), regarding EBV/VCA-IgG (A1) and CMV-IgG (B1). *p* ≤ 0.05 was considered statistically significant. IFN-γ, interferon-gamma; GM-CSF, Granulocyte-macrophage colony-stimulating factor; TNF, tumor necrosis factor-alpha; RR-SMS, relapsing-remitting stable phase; PP-MS, primary progressive; CMV, Cytomegalovirus; EBV/VCA, Epstein Barr viral capsid antigens.

Significant positive correlations were observed between EBV/VCA-IgG antibody titers and pHERV-W_env 486-504_ cytokine-positive CD19+ B-cells in PP-MS patients (**Figure 5A**), particularly for IFN-γ and GM-CSF (**Figure 5A.1**). Conversely, a strong negative correlation was found between IFN-γ, GM-CSF and TNF-α levels in RR-SMS patients, as well as between GM-CSF, TNF-α and CMV-IgG antibody titers in PP-MS patients (**Figures 5B, B.1**). No significant correlation was detected with syncytin-1_env 486-500_ cytokine-positive CD8+ and CD19+ cells in either HC or MS populations.

## 4 Discussion

This study investigates the serological response to antigenic peptides derived from pHERV-W and syncytin-1 envelope proteins across different stages and types of MS. It also employs discriminant analysis to evaluate whether integrating clinical variables with humoral response data against other MS-associated viral factors can enhance the prediction of clinical course.

The results show that IgG ratio titers against pHERV-W_env 486–504_ */*syncytin-1_env 486–500_ are elevated in all MS patients compared to HCs. Notably, patients with progressive MS have higher IgG ratio responses against these peptides than those with relapsing forms (RR-SMS and RR- AMS), suggesting that pHERV-W may contribute to MS progression. Furthermore, the higher antibody ratio titers against pHERV-W_env 486-504_ and syncytin-1_env 486-500_ in RR-AMS patients indicate a potential link with MS exacerbation. However, an imbalance in gender and age distribution within the sample may have influenced these results, although no significant differences between genders or ages were found.

The study aligns with previous research indicating that pHERV-W env proteins can trigger inflammatory responses and neurodegeneration in MS by promoting immune cell infiltration and activation, inhibiting remyelination, and increasing pro-inflammatory cytokine release from lymphocytes, monocytes, macrophages, and microglia (24–26). Furthermore, *in vitro* and animal studies have shown that anti-pHERV-W antibodies recognize myelin oligodendrocyte glycoprotein and that immunization with pHERV-W, when combined with the MOG_35–55_ peptide, can activate experimental autoimmune encephalomyelitis (EAE) (18, 27). Conversely, compared to patients with other demyelinating disorders, including neuromyelitis spectrum disorder (NMOSD) and myelin oligodendrocytes glycoprotein-antibody disease (MOGAD), MS patients exhibit a stronger antibody response to pHERV-W and syncytin-1 (28). Consistent with these findings, previous studies have shown that pHERV-W env gene expression levels were higher than syncytin-1 in RR-MS and PP-MS patients (29).

Additional aim of this study was to explore T-lymphocytes, B-cells, along with their associated cytokine response responses after stimulation with syncytin-1_env 486–500_ and pHERV-W_env 486–504_. MS has been traditionally linked to CD4 T-cells, due to genetic associations with the MHC class II region (30). However, CD8 T-cells and CD19 B-cells are also crucial in the disease’s humoral response (31, 32). TNF-α, a major mediator of inflammation in MS (33), was significantly increased in both CD19+ B-cells and CD8+-T cells after exposure to pHERV-W_env 486–504_, especially in RR-SMS and PP-MS patients and was higher compared to HCs. Additionally, pHERV-W_env 486–504_ also triggered increased levels of IFN-γ, with the peak response observed in PP-MS patients. IFN-γ, which typically rises before MS relapses, plays a crucial role in immune response and viral infections (34–36). Cytometric analysis revealed a significant increase in GM-CSF levels, a cytokine linked to inflammation in MS (37), produced by CD8+ T-cells after exposure to pHERV-Wenv 486–504 in RR-SMS and PP-MS patients. RR-MS patients also showed higher GM-CSF produced by CD19+ B-cells. Moreover, was detected significant increases in IL-17 levels produced by CD8+ T-cells and CD19+ B-cells in RR-MS patients following pHERV-W_env 486–504_ exposure. IL-17 plays crucial role in MS by activating CNS-resident cells, leading to neuron hyperexcitability and increased cytokine and chemokine production, which in turn triggers neuroinflammation (38, 39). Additionally, IL-17A is known for promoting the migration of human CD4+ T-cells across the blood-brain barrier (40), potentially explaining the reduced CD4+ response observed in the peripheral blood of MS patients in this study. Therefore, the increased anti-pHERV-W humoral response and the triggering of cytokine release by it suggest its potential role in monitoring peripheral inflammation in MS.

Interestingly, we found a strong correlation between pHERV-W_env 486–504_-induced cytokine production and EBV and CMV titers in MS patients. MS has been linked to various viruses, including HHV-6 and EBV (3, 41), which have been documented to activate HERV-W and MSRV genes (42–46). Notably, the EBV envelope protein gp350 is known to activate syncytin-1 and MSRV gene transcription (8). EBV infections can activate MSRV-associated genes, bypassing the usual inhibitory effects of viral genome methylation (43, 44). A Spanish study also found that EBV viral load changes correlate with pHERV-W gene expression in RR-MS patients, suggesting EBV might be an early trigger for MS and chronic neuroinflammation (47). Conversely, CMV seropositivity appears to lessen the severity of EBV responses and may reduce MS risk (48). This immune competition between EBV and CMV could be protective. Some evidence suggests that CMV seropositivity in mothers may lower MS risk in their children, potentially by modulating the inflammatory response triggered by EBV and influencing pHERV-W expression (49, 50).

The interaction between HERV-W and other viruses in MS is complex. HERV-W proteins may modulate immune responses triggered by other viruses, influencing inflammatory pathways and disease outcomes. The structural similarities between viral proteins and HERV-W proteins can lead to cross-reactive immune responses, which may contribute to the autoimmune aspects of MS. Furthermore, developing a discriminant model that integrates demographic and clinical factors with serological data could refine the classification between MS types, improve personalized treatment, and help identify misclassified cases.

Understanding these interactions could provide valuable insights into disease mechanisms and potential therapeutic targets. In conclusion, this study highlights that elevated humoral responses against pHERV-Wenv and syncytin-1 in MS patients, particularly those with progressive forms, support the involvement of pHERV-W in MS pathogenesis. The association of pHERV-W with disease exacerbation, coupled with the development of discriminant models based on clinical and serological data, enhances our understanding of MS progression and identifies potential biomarkers for disease management.

## Data Availability

The raw data supporting the conclusions of this article will be made available by the authors, without undue reservation.
