# Supplementary figures and images for: Immune response profiling of HERV-W envelope proteins in multiple sclerosis: potential biomarkers for disease progression
Source: Front Immunol. 2025 Jan 9;15:1505239. doi: 10.3389/fimmu.2024.1505239 (PMC11754046; doi:10.3389/fimmu.2024.1505239)

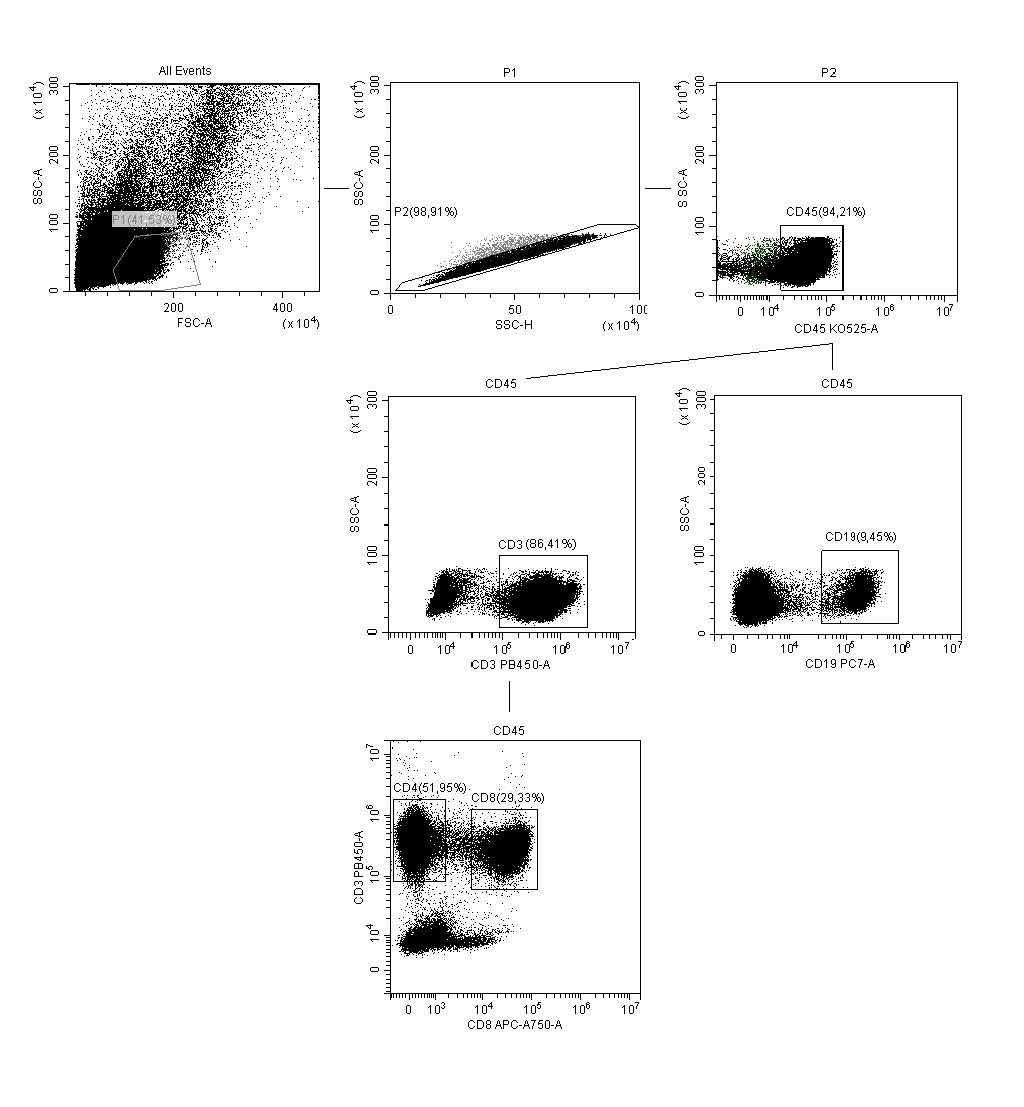

Supplement: Supplementary file 1 [file Image1.jpeg]

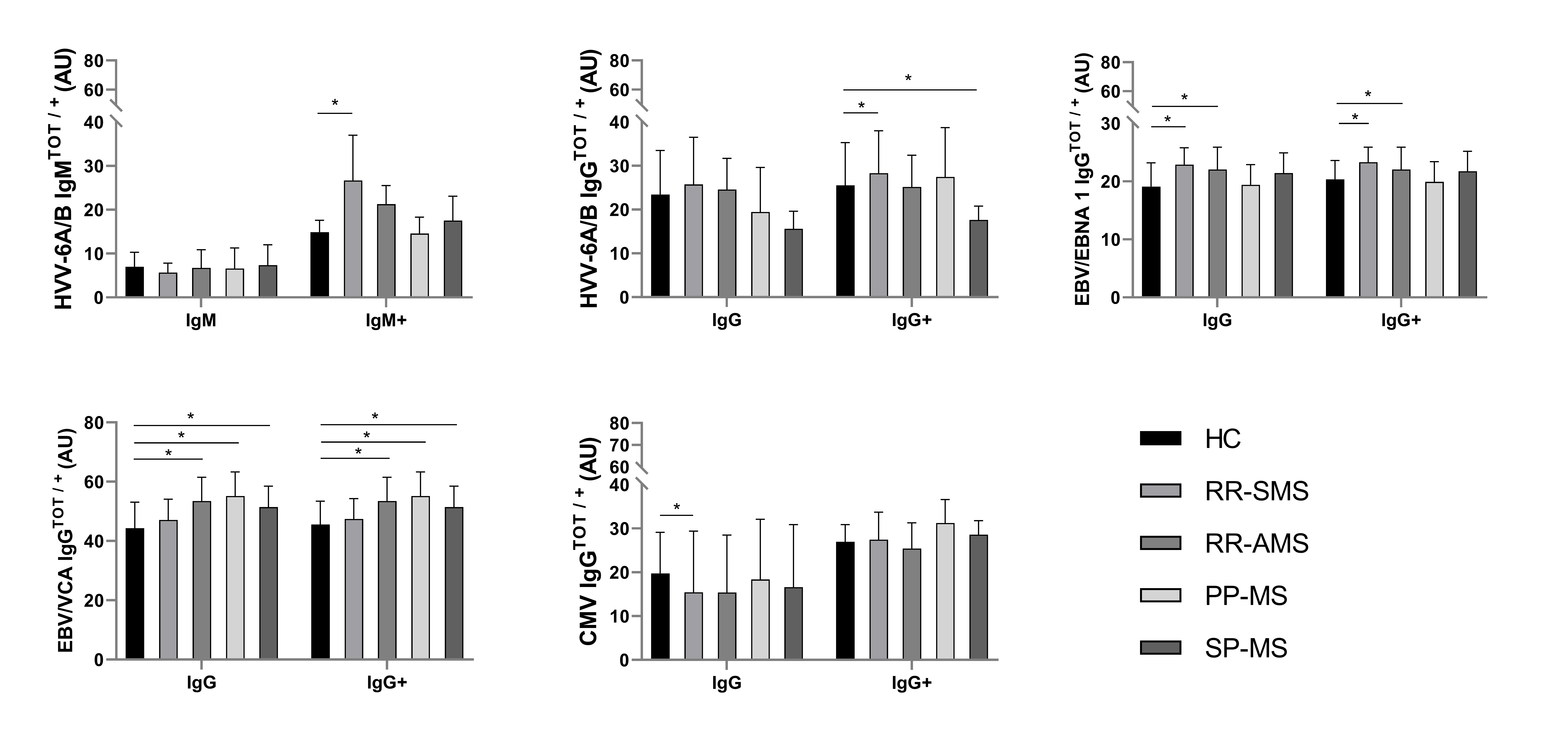

Supplement: Supplementary file 2 [file Image2.tif]
