# Supplementary material for: Immune response profiling of HERV-W envelope proteins in multiple sclerosis: potential biomarkers for disease progression
Source: Front Immunol. 2025 Jan 9;15:1505239. doi: 10.3389/fimmu.2024.1505239 (PMC11754046; doi:10.3389/fimmu.2024.1505239)
Supplement: Supplementary file 3 [file Table1.docx]

Supplementary Material

**
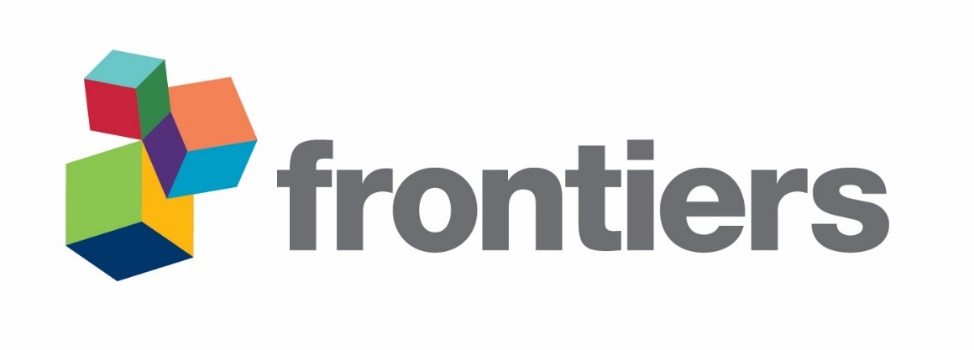
**

**Supplementary Figure 1.** **Supplementary Data**

Strategy for the analysis of lymphocyte subpopulations from PBMCs.

**Supplementary Figure 2. Supplementary Data**

Comparison of total IgG/IgM level and comparison of patients exceeding the IgG/IgM cut-off threshold for antibody titers to HHV-6A/B IgM and IgG, EBV/EBNA-1, EBV/VCA, and CMV IgG in patients and controls. The figure displays the level of total IgG (or IgM) antibodies in patients and controls, as well as the proportion of IgG (or IgM) level above the established cut-off (IgG+/IgM+). IgM/IgG concentrations were quantified using an ELISA-based specific kit. The figures display the median with IQR. Mann–Whitney test and Kruskal–Wallis with Dunn’s test. *p* ≤ 0.05 was considered statistically significant. * Indicate significant differences compared to HCs.

**Supplementary Table 1.** **Supplementary Data**

**Table 1.**

Standardized discriminant coefficients for the categorical variables studied.

|  | Function | |
| --- | --- | --- |
|  | 1 | 2 |
| *p*HERV-W/Syncytin-1 | 0.341 | -0.007 |
| AGE | -0.180 | -0.665 |
| EDSS | 0.737 | 0.199 |
| Age Disease (Onset) | 0.679 | 0.782 |
| Disease Duration | 0.429 | -0.320 |
| HHV6 IgG | -0.143 | 0.299 |
| HVV6 IgM | 0.065 | -0.166 |
| EBNA1 IgG | -0.100 | -0.247 |
| VCA IgG | 0.048 | 0.330 |
| CMV IgG | -0.166 | 0.272 |
